# Molecular Dynamics Simulations of Influenza A Virus NS1 Reveal a Remarkably Stable RNA-Binding Domain Harboring Promising Druggable Pockets

**DOI:** 10.3390/v12050537

**Published:** 2020-05-14

**Authors:** Hiba Abi Hussein, Colette Geneix, Camille Cauvin, Daniel Marc, Delphine Flatters, Anne-Claude Camproux

**Affiliations:** 1 Université de Paris, BFA, UMR 8521, CNRS, ERL U1133, Inserm, F-75013 Paris, France; hiba.abihussein@gmail.com (H.A.H.); colette.geneix@univ-paris-diderot.fr (C.G.); camille.cauvin@gmail.com (C.C.); anne-claude.camproux@univ-paris-diderot.fr (A.-C.C.); 2 Equipe 3IMo, UMR1282 Infectiologie et Santé Publique, INRAE, F-37380 Nouzilly, France; 3 UMR1282 Infectiologie et Santé Publique, Université de Tours, F-37000 Tours, France

**Keywords:** influenza A virus, NS1, molecular dynamics, druggability, druggable pockets

## Abstract

The non-structural protein NS1 of influenza A viruses is considered to be the major antagonist of the interferon system and antiviral defenses of the cell. It could therefore represent a suitable target for novel antiviral strategies. As a first step towards the identification of small compounds targeting NS1, we here investigated the druggable potential of its RNA-binding domain since this domain is essential to the biological activities of NS1. We explored the flexibility of the full-length protein by running molecular dynamics simulations on one of its published crystal structures. While the RNA-binding domain structure was remarkably stable along the simulations, we identified a flexible site at the two extremities of the “groove” that is delimited by the antiparallel α-helices that make up its RNA-binding interface. This groove region is able to form potential binding pockets, which, in 60% of the conformations, meet the druggability criteria. We characterized these pockets and identified the residues that contribute to their druggability. All the residues involved in the druggable pockets are essential at the same time to the stability of the RNA-binding domain and to the biological activities of NS1. They are also strictly conserved across the large sequence diversity of NS1, emphasizing the robustness of this search towards the identification of broadly active NS1-targeting compounds.

## 1. Introduction

Influenza A viruses remain a permanent threat to human and animal health. It is estimated that, each year, one billion humans are infected with seasonal influenza, causing 3–5 million severe cases [1] and resulting in an excess mortality of 290–650 thousand deaths per year [2]. In addition to these seasonal viruses, the hundreds of cases of human infection with H5N1 and H7N9 avian influenza viruses that have been recorded since 2000 (combined death toll above 1000) illustrate the threat that these emerging viruses pose to human health, should they become pandemic by gaining the ability to transmit between humans. While vaccination can to some extent control the incidence and reduce the severity of seasonal influenza, its utility in the preparedness against future pandemics is questionable, given the ~6-month schedule of the current manufacturing process. Therefore, antiviral therapies are of paramount importance in the treatment of severe influenza-related respiratory infections, both in the case of seasonal influenza and in the event of a new pandemic.

Adamantanes, the first generation of influenza antivirals, targeting the M2 ion channel, are now essentially obsolete because the circulating viruses have become resistant to them [3], and, currently, the therapeutic options are limited to essentially two neuraminidase inhibitors—oseltamivir and zanamivir—along with the recently approved viral-polymerase inhibitor baloxavir marboxil [4,5,6]. None of these molecules are immune to the development of resistance [7], and there is therefore a pressing need to enrich our assortment of antivirals, both to be able to respond to emerging resistance and to design combination therapies that could outperform the single molecule treatments. Any combination therapy should target more than a single viral protein and more than one step of the viral cycle.

Influenza virus non-structural protein NS1 stands as one of the promising targets [8]. This multifunctional RNA-binding protein is considered to be the main actor of the virus to antagonize the antiviral response of the host [9,10,11,12,13]. It is highly expressed in the infected cell, where it exhibits several pro-viral activities [14,15]. This 230-residue protein, encoded by the smallest of the eight RNA segments that compose the viral genome, is a homodimer composed of two structured domains. The RNA-Binding Domain (RBD) is a compact, obligate dimer formed by residues 1–73 of the two symmetric polypeptide chains, each contributing three alpha helices. A flexible 10-residue linker region connects each RBD monomer to the globular Effector domain (residues 85–202), which is a non-obligate dimer. The C-terminal tail (residues 203–230) is likely an intrinsically disordered region [16]. The multiple activities of NS1 may to some extent rely on its conformational plasticity [17,18]. Through its interaction with RNAs [19] and several viral and cellular proteins, NS1 is involved in several stages of the viral cycle. Nevertheless, it has been possible to engineer recombinant influenza viruses that are devoid of NS1 [20]. These viruses show a dramatically reduced replication potential. A similar phenotype is observed when the RNA-binding property of NS1 is abolished by critical substitutions in the RBD [9,12,13,21], supporting the potential interest of antiviral strategies targeting the RNA-binding interface of NS1.

Some experimental studies have explored influenza virus NS1 protein as a therapeutic target: in vitro or cell-based screens were carried out to identify small compounds that block one of the measurable activities of NS1 in vivo or in vitro: its detrimental effect on yeast growth [22,23,24], its inhibition of gene expression [25] or its binding to dsRNA in vitro [26]. However, the binding site of these compounds was not always determined. Alternatively, in silico approaches were used to dock small compounds onto NS1′s structure. Darapaneni et al. identified the most conserved residues in NS1 amino-acid sequences and searched for putative ligand binding sites using Q-SiteFinder [27], while Ahmad et al. followed a similar strategy to dock chemical compounds from *Azadirachta indica* leaves onto NS1′s structure [28]. Some of the small compounds identified by these different approaches were found to inhibit viral replication in cell-based models. On the other hand, assessing the druggability potential of a target is a key step towards successful discovery [29]. Combining sequence analysis and druggability prediction algorithms, Trigueiro-Louro et al. systematically probed in silico the druggability of NS1′s Effector domain [30]. All of these studies show the promise of NS1 as a therapeutic target.

In the present work, we sought to systematically assess the druggability of NS1 by taking into account the inherent flexibility of its structure. More specifically, we focused on the RNA-binding interface of its RNA-binding domain (RBD), based on the rationale that invalidating the functionality of this domain dramatically reduces both the replication potential of the virus and its pathogenicity. We studied the stability of NS1′s structure through a Molecular Dynamics (MD) approach using the three-dimensional structures of both the full-length protein and its isolated RBD. We evaluated the flexibility of the structures and their ability to form cavities or pockets, specifically within the RNA binding interface that encompasses the groove formed by the two antiparallel helices α2 and α2′. We estimated pockets in the groove along the dynamics simulations and studied their druggability, i.e., their potential to bind drug-like ligands, as assessed by the PockDrug-Server [31]. We characterized and clustered all the groove-pockets observed during the MD simulation, in terms of frequency, accessibility, physico-chemical and geometrical properties. We also examined the conservation of the residues involved in the different classes of pockets in the RNA-binding interface. This allowed us to identify promising druggable pockets and to confirm the potential of the RBD as a drug target.

## 2. Materials and Methods

### 2.1. NS1 Three-Dimensional Protein Structures

**Figure 1:**
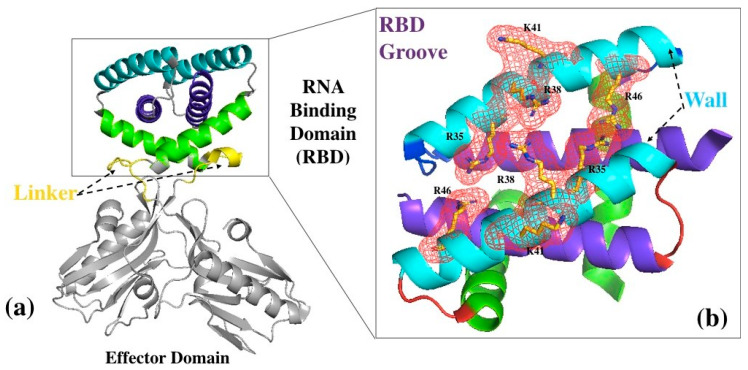
Crystal structure of the full-length protein. (**a**) Ribbon representation of the dimeric full-length protein with its two domains (Protein Data Bank (PDB) access number 4OPA): the RNA-binding domain dimer (framed in black) comprises six symmetry-related α-helices (α1 and α1′ in deep blue, α2 and α2′ in cyan, α3 and α3′ in green). The two domains are connected by a linker (yellow) (**b**) zoom-in on the RNA-binding domain and its groove, viewed from the top with respect to orientation in (**a**); the antiparallel α2-helices (cyan, residues 30–50) form the walls of the groove, while helices α1 and α1’ (deep blue, residues 3–24) form its bottom. Helices α3 and α3’ (green, residues 54–70) connect the RNA-Binding Domain (RBD) to the Effector domain via the linker. Residues R35, R38, R46 and K41 from the α2 helices are represented in mesh (red).

The two 230-residue chains that make up the homodimeric structure of NS1 are arranged in two domains: RBD and Effector domain (Figure 1a). The RBD is an obligate dimer involving residues 1–73 of the two chains (A and B), each one contributing three α-helices (α1/α1′, residues 3–24; α2/α2’, residues 30–50; and α3/α3′, residues 54–70). Each peptide chain is connected via a flexible linker region to the effector domain (residues 81–230). The orientation of the two effector domains relative to each other and to the RBD can accommodate some variations, notably in relation with the length of the linker [17,32]. In the present study we defined the groove as the concave surface formed by the α-helices 2, 2′ and 1, 1′ of the RBD. Residues 5–19 of the α-helices 1 and 1′ form the bottom of the groove, while residues 29–46 of the α-helices 2 and 2′ that form its rim and walls also make up the RNA-interaction surface, which is diametrically opposed to the linkers and effector domains. Highly conserved, positively-charged amino acids in the middle of this interface (Arg35, Arg37, Arg38 and Lys41) make several direct or water-mediated hydrogen bonds and electrostatic interactions with both strands of the dsRNA sugar-phosphate backbone (Figure 1b). Several other residues outside this positive patch (Thr5, Asp29, Asp34, Ser42 and Thr49) also contribute indirectly to the RNA interaction by extending the RNA-stabilizing network of hydrogen bonds [33]. From now onwards, we will use the one letter code for the amino acid residues.

The structure of each individual domain of NS1 has been solved for several variants of the protein [33,34,35,36,37,38,39,40,41]. In addition, the structure of complexes associating the effector domain with some of its binding partners has been determined [42,43]. Finally, the structure of the full-length protein was solved for three variants and mutants thereof [17,32,44], revealing the intrinsic flexibility of NS1 that allows the monomeric Effector domains to adopt three distinct conformations (“open”, “semi-open” or “closed”) relative to the RBD.

As a starting model of the three-dimensional structure, we chose the PDB structure 4OPA [32]. This homodimeric structure composed of two 225-residue chains was solved from a recombinant NS1, originating from the avian influenza virus A/blue-winged teal/MN/993/1980 (H6N6). Its amino-acid sequence, close to that of most H5N1 viruses, is representative of NS1 prototype p1.0, which is the most prevalent among avian influenza viruses [45]. Secondly, among the three full-length structures available at the beginning of this study (PDB access numbers 3F5T, 4OPH and 4OPA), 4OPA has the best resolution (2.7 Å). Finally, it harbors a shortened linker, resulting from the removal of amino-acids 80-84 that was designed to match the identical deletion that appeared in 2000 in field isolates of H5N1 avian influenza viruses. In addition, the recombinant protein 4OPA harbors the R38A-K41A double substitution that was designed in order to prevent aggregation of the full-length recombinant protein [32,44]. In structure 4OPA the Effector domains are in the “closed” conformation.

### 2.2. Molecular Dynamic Protocol and Analysis

In the structure 4OPA, we first reversed beforehand the R38A-K41A double substitution to its wild-type sequence R38-K41. From this reversed structure, named “FL” (Full Length), we also extracted the RBD structure by removing residues after position 75 from the two chains A and B of the dimer. The resulting structures were first treated with ProPka [46] to assign protonation states at pH 7, and to build missing lateral chains by ensuring that new atoms are not rebuilt too close to existing atoms. MD simulations were carried out with Gromacs v. 5.0.2 [47,48], using the Amber99SB force field under periodic boundary conditions. All structures were simulated as immersed in a water box of the TIP3P water molecules model (14 Å-thick layer of water). Non-bonded interactions were truncated at a cut-off distance of 10 Å for the electrostatic twin-range cut-off and the Van der Waals cut-off. The energy of the system was minimized over 2000 cycles, using the steepest descent algorithm for energy minimization. Then, counter-ions were added to neutralize the system and a second minimization was performed applying the particle-mesh Ewald (PME) method for the evaluation of long-range electrostatic interactions. The minimization converges at a maximal force of 1000 kJ/mol/nm for the first minimization and 2000 kJ/mol/nm for the second one. Each MD simulation was preceded by a 1 ns period of heating/equilibration during which harmonic restraints were imposed on the atomic positions of the protein and counter-ions. Each simulation was performed at a constant temperature (300 K) and pressure (1 atm), the isothermal-isobaric (NPT) ensemble, coupling the system to a heat bath, using the Berendsen algorithm. The LINCS algorithm was applied to all bond lengths to constrain them that allowed using an integration time step of 2 fs. Two 50 ns and one 40 ns simulations were run for FL, and three 50 ns simulations were run for RBD. MD simulations were visualized using Visualized Molecular Dynamics 1.9.2 [49]. A snapshot (conformation) of the structure was saved every 10 picoseconds, yielding a set of 5000 conformations for each trajectory. Using GROMACS tools, several properties were analyzed along the simulations to validate their quality and stability, including Root Mean Square Deviation (RMSD), gyration radius, secondary structure maps, principal component analysis (PCA), and angles χ1 χ2 of residues of interest.

These simulations were subsequently concatenated in two trajectories of 140 and 150 ns for the FL and RBD, respectively. Root Mean Square Fluctuation (RSMF) were analyzed to identify the flexible residues. The most representative FL and RBD conformations were selected by clustering these trajectories based on an RMSD similarity threshold of 2 Å between all conformation pairs.

### 2.3. Druggable Pocket Estimation and Analysis

#### 2.3.1. Groove-Pocket Localization

To study NS1 pockets, conformations were sampled at one nanosecond intervals from FL and RBD trajectories, yielding two sets of 140 and 150 conformations. These two sets were named FL-conf and RBD-conf, respectively. We estimated surface pockets from FL-conf and RBD-conf, using Fpocket, a geometry-based method that investigates all cavities of a protein independently of any ligand information [50]. Next, we selected the pockets located within the groove, i.e., those including at least one residue from each of the α2 helices (α2/α2′, residues 30–50) that delimit the groove.

#### 2.3.2. Groove-Pocket Analysis

In the present study, we excluded the smallest pockets involving fewer than 14 residues, considering that they mainly correspond to decoy pockets [31,51]. Moreover, pockets including at least 14 residues generally exceed the 600 Å3 volume that is appropriate to bind drug-like molecules [52]. The corresponding pockets will be referred to as groove-pockets. As a first step, pockets were described using 75 descriptors (encompassing the 52 PockDrug descriptors [53]), which include (i) physicochemical properties such as atom composition, residue composition, hydrophobicity, polarity and charge and (ii) geometrical ones such as the numbers of pocket atoms and residues, volume and sphericity.

Although the different descriptors provided complementary information, some of them can be highly correlated, and we therefore carefully selected a restricted set of descriptors to obtain a balanced information between geometrical and physicochemical properties. We removed descriptors with null variance and for each subset of correlated descriptors (Pearson correlation parameter > 0.85), and only one representative descriptor was retained. In addition to these descriptors, Pockdrug [31] provides a prediction of the pocket druggability; groove-pockets were defined as druggable if their Pockdrug druggability score exceeded 0.5. To visualize the space sampled by the groove-pockets, we performed a PCA based on the selected physicochemical and geometric descriptors, using the FactoMineR package in R package (v. 3.1.1). Groove-pockets were then explored in a lower dimensional space spanning the most informative view, according to their variability, and finally clustered according to their geometrical and physicochemical profiles.

A hierarchical classification based on the weighted Euclidean distance was performed, building embedded partitions through the stepwise aggregation of the pocket pairs and then the pair clusters according to the Ward metric [54]. The resulting tree representation, which visualizes the proximity of the pocket in terms of the selected descriptors, allows for the identification of the main pocket clusters. Finally, groove-pockets were analyzed in terms of the accessibility, conservation and importance in the NS1 structure of their residues.

## 3. Results

### 3.1. NS1 Structure Analysis

#### 3.1.1. Dynamic Properties of NS1 Structure

The FL and RBD simulations, which were run under the physiologically relevant conditions of 300 K and pH 7, were observed to sample largely the NS1 conformational space. The average RMSD below 3 Å and the well-conserved secondary structures (Supplementary Figures S1 and S2) confirm the stability of NS1 dimer, both in the FL protein and in the RBD.

**Figure 2:**
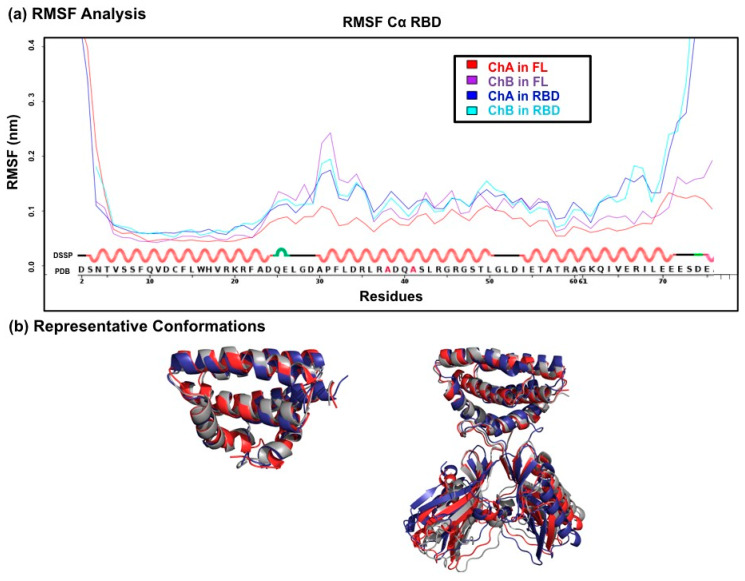
Flexibility of the RBD (**a**) The RMSF curves were computed on the Cα of residues 1–73 of each chain of the dimeric RBD (named ChA and ChB). The RMSF of the two chains were computed on the concatenated trajectories of the RBD (blue and cyan curves) or of the FL (red and violet curves). The DSSP secondary structure assignation of the sequence (below) shows red helices linked by loops. (**b**) Shown are the superimposed representative conformations of the three main clusters of conformations obtained by clustering RBD-conf (**left**) and FL-conf (**right**).

For each of the concatenated trajectories (RBD and FL), we plotted the Root Mean Square of Fluctuation (RMSF) of the alpha carbons of the A and B chains of the RBD in order to evaluate the inherent flexibility of the structures (Figure 2a). This unexpectedly revealed a region of high flexibility centered on residue 31 and encompassing the α1–α2 connecting loop and the N-terminal third of helix α2. By contrast, the lowest level of flexibility was observed in helix α1, which is not unexpected since helix α1 interacts with residues from α-helices 2, 2′ and 3, 3′. The C-terminal extremity of helix α3 tends to unfold slightly during the simulations of the RBD but not of the FL, as confirmed by the lower RMSF values observed in the FL simulations. This likely results from the fact that the end of helix α3, unconstrained in the RBD form, is connected to the Effector domain in the FL protein. In general, we observed that the presence of the Effector domain tends to stabilize the RBD. Three main clusters of the RBD (or FL) conformations were obtained as a result of the conformation clustering using an RMSD similarity threshold of 2 Å. Representative conformations of the three main clusters are illustrated in Figure 2b.

The interactions between the two monomers mainly involve residues from the α-helices 1 and 1′ at the center of the RBD, with the added contribution of some residues in α-helices 2 and 2′. For instance, residue R35 of α2 (and symmetrically α2′) makes a hydrogen bond and a salt bridge with residue D12 of α1′ (and α1, respectively). At the interface between the two monomers (dimerization zone), the hydrogen bonds and the intra- or inter-monomer salt bridges are stable over the duration of the simulation, which likely reflects the large contribution of the dimeric assembly to the stability of the RBD.

#### 3.1.2. Pocket Identification

Out of the 140 and 150 conformations from FL-conf and RBD-conf, Fpocket identified a total of 3578 pockets: 2301 in the FL-conf and 1277 in the RBD-conf, corresponding to an average number of pockets per conformation of 16.44 and 8.51 for FL and RBD, respectively.

We identified 1076 pockets in RBD-conf after excluding linker-specific pockets, and 867 pockets in FL-conf after excluding linker- and ED-specific pockets. The higher pocket number in RBD-conf likely results from the absence of the ED, which artificially increases the accessibility of the α3 helices. By further reducing our focus to the groove region, we identified 268 and 302 pockets in FL-conf and RBD-conf, respectively, totaling 570 pockets among the 290 conformations. After exclusion of the pockets involving fewer than 14 residues (see methods), we obtained a total of 323 pockets located in the groove, named groove-pockets.

### 3.2. Groove-Pocket Analysis

#### 3.2.1. The Groove Is Most Often in a Druggable State

The similar numbers of pockets identified in RBD-conf and FL-conf (164 and 159, respectively) confirm the stability of the groove region and its ability to form potential binding pockets regardless of the presence or absence of the Effector domain. Most of the groove conformations (84%) contained exactly one groove-pocket, while 13% (21 FL and 19 RBD) exhibited two pockets and 3% had none (4 FL and 6 RBD).

Each pocket being associated with a druggability score, we explored the druggability of the groove over the course of the FL and RBD trajectories. We considered the groove as druggable if it included at least one druggable pocket (PockDrug-predicted druggability score above 0.5).

**Figure 3:**
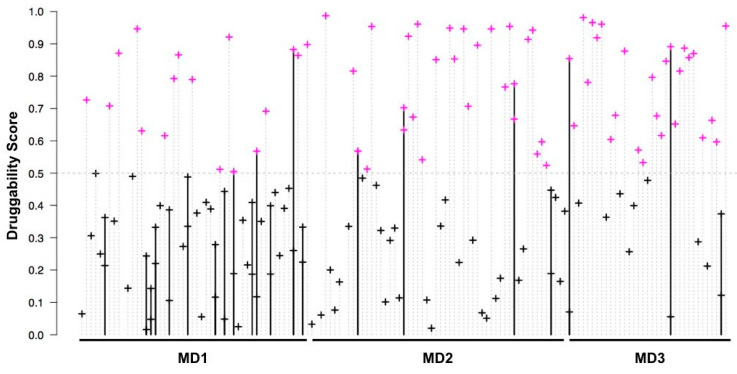
Druggability of the 159 pockets identified in the groove region for the 140 sampled FL conformations, obtained on three independent dynamics. Vertical lines stand for the consecutive conformations that were sampled (at 1 ns intervals) along each of the three Molecular Dynamics (MD) trajectories, with the terminating cross representing the druggability score of its groove pocket(s). Druggable pockets (druggability score ≥ 0.5) are in violet, the others are in black. Bold lines (with two dots) represent the 21 conformations that contained two groove pockets.

Over both FL and RBD simulations, we observed a permanent fluctuation (at one nanosecond intervals) between druggable and non-druggable states, as shown in Figure 3, which illustrates the evolution of the groove’s druggability along the FL dynamic simulation. This is coherent with the flexibility of this region and confirms again that the presence of the Effector domain minimally impacts the druggability of the groove. In ~60% of the sampled timepoints (76/140 FL and 98/150 RBD), the groove exhibited a druggable conformation. Of note, we observed that the groove in the initial structure (PDB 4OPA) is in a non-druggable conformation, emphasizing the interest of taking into account the flexibility of a protein to predict its druggability.

#### 3.2.2. Characterization of the Groove Pockets

**Figure 4:**
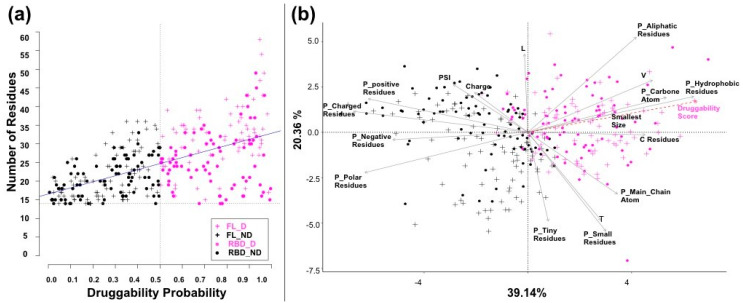
Pocket druggability and principal component analysis (PCA). (**a**) The scores of the 323 groove-pockets plotted against their number of residues (correlation value of 0.57, *p*-value < 2.2 × 10^−16^). (**b**) Two-dimensional projection of the 17 descriptors onto the first two PCA axes. The druggable pockets (druggability score ≥ 0.5) are in violet, and the others are in black. Crosses correspond to FL pockets, and dots correspond to the RBD pockets.

The 323 groove-pockets that we identified along the dynamics have variable druggability scores. These scores significantly increased with the number of residues involved (correlation value of 0.57, *p*-value < 2.2 × 10^−16^, Figure 4a): less druggable pockets tend to involve fewer residues (14 to 23) than druggable ones (up to 55 residues), in agreement with previous studies [31,53]. We next attempted to evaluate the relation between the PockDrug-predicted druggability score and 17 selected groove-pocket descriptors. Of these, three relate to the pocket geometry and shape, eleven are dedicated to physicochemical properties such as the proportion of hydrophobic or aliphatic residues and three correspond to the presence of amino acids (T, L, V) (see Supplementary Table S1).

A PCA was run to analyze the contribution of the selected descriptors to the druggability of the groove-pockets. The descriptors, along with druggability score, were projected onto the first plane of a PCA, which captures ~60% of the pocket variability (Figure 4b). Of note, we observed that the distributions of the FL- and RBD-pockets on the first PCA plane are very similar, reflecting their parallel properties.

The first axis, capturing 40% of the variability, discriminates druggable pockets from less druggable ones, by combining descriptors of size and hydrophobicity. In agreement with previous studies [31,53], high druggability scores correspond to large pockets containing hydrophobic and aliphatic residues, which notably include valine residues. Conversely, lower druggability scores correspond to small, spherical pockets that involve polar and charged residues (Charge and P_charge-value, P_negative_residues). The second axis, capturing 20% of the variability, opposes small, tiny or threonine residues to aliphatic and leucine residues.

**Figure 5:**
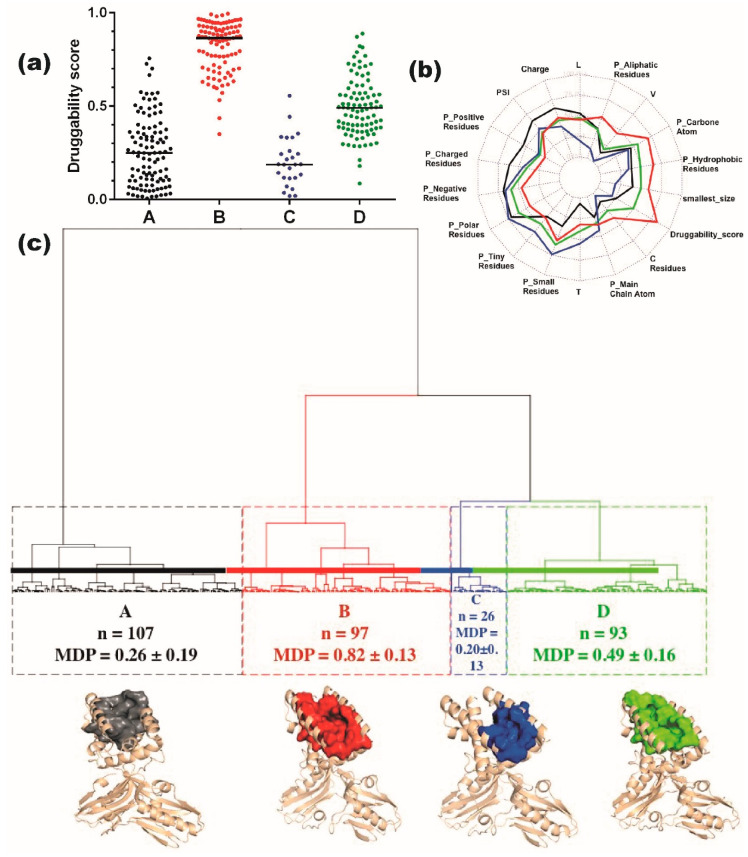
Groove-pocket classification: (**a**) The four groups A, B, C, and D of 323 groove-pockets, with their druggability scores. (**b**) Radar plot showing the correlation between each cluster and 18 descriptors. Colored lines correspond to the four groups of pockets (A, B, C, D, in black, red, blue and green, respectively). (**c**) Hierarchical clustering, with a surface rendering of a representative pocket from each of the four groups. The number of pockets, the mean druggability score (MDP) and its corresponding standard deviation are given for each class. The representative structures of NS1 (cartoon format) and of the groove-pockets (surface rendering) for each cluster are also shown.

To further extend our analysis of the 323 “groove-pockets”, we clustered them based on the similarity of their seventeen descriptors. This classification yielded four main groups, denoted as A, B, C and D, which comprised 33%, 30%, 8% and 29% of the pockets, respectively (Figure 5a). The four groups each contain pockets from both FL-conf and RBD-conf; their 17 descriptors and the druggability score are depicted on the radar plot (Figure 5b).

Group A contains 107 pockets of low druggability score (0.26 ± 0.19), characterized by a small volume and a higher occurrence of charged and positive residues. Group C contains 26 low-druggability pockets (0.20 ± 0.13) that involve small, nonpolar, few charged residues, with less L and V but more T residues than in group A. Group D includes 93 pockets of medium druggability score (0.49 ± 0.16), while group B comprises 97 highly druggable pockets (0.82 ± 0.13). The average druggability scores of groups C, A, D, and B increase in parallel with the average number of residues and with the involvement of carbon atoms, aliphatic, hydrophobic residues and valine residues (Figure 5b). A representative protein-pocket structure of each cluster is shown in Figure 5c.

### 3.3. Residues Involved in Groove-Pockets

**Figure 6:**
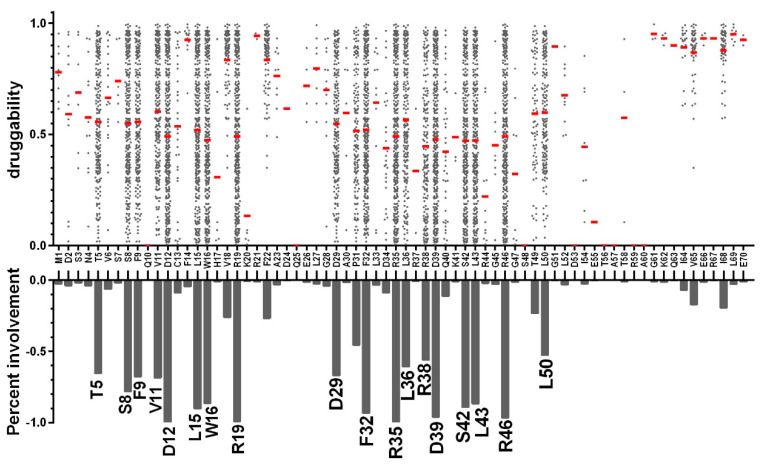
Residues involved in the 323 groove-pockets. For each of the RBD residues, each dot in the upper graph depicts one groove-pocket in which it is involved, with its druggability score (y-scale); the mean bar (red) represents the average druggability score of all the pockets in which a given residue is involved. The lower graph shows the percentage of groove-pockets in which each residue is involved.

Since the druggability of the pockets mainly results from the properties of the residues involved, we examined the pockets by focusing more specifically on the RBD residues (positions 1–70), even if a few pockets (5/323, mainly from the FL-conf) involve residues from the linker region. For each of these 70 residues, Figure 6 plots the druggability score of all the groove pockets in which it is involved.

**Figure 7:**
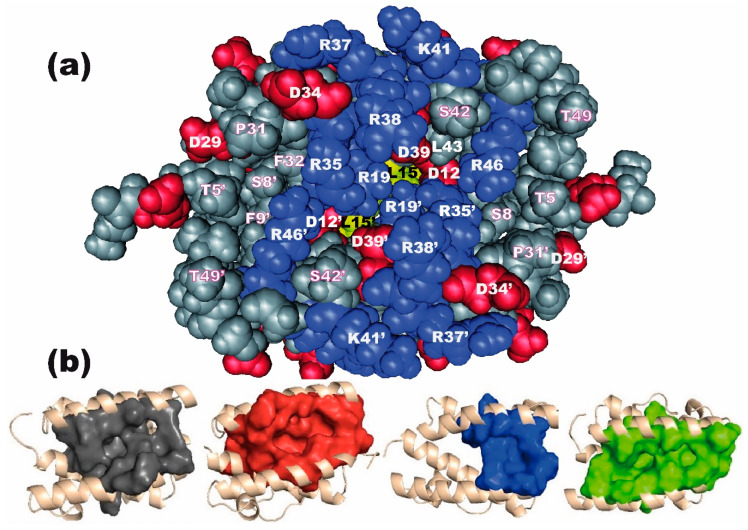
Top view of the groove. (**a**) Space-filling representation of the RBD using software Cn3D. Viewed from above the two nearly coplanar α-helices 2 and 2′, the structure is that of the 1918-Brevig Mission virus (PDB access N°2N74), which, unlike 4OPA, has the wild-type residues at positions 38 and 41. Charged residues (red: acidic residues Asp, Glu; blue: basic residues Arg, Lys, His) are highlighted, as well as the two L15 residues at the bottom of the groove (yellow). Most of the indicated residues belong to α-helices 2 and 2′ (residues P31-T49). (**b**) Surface rendering representative of the four groups of pockets (in the order A, B, C, D).

We first observed that, on average, the druggability score increases with the number of residues involved (Figure 4a). Of the 70 residues in the RBD, eleven (S8, D12, L15, W16, R19, F32, R35, D39, S42, L43, and R46) are observed in more than 75% of the 323 pockets, regardless of their druggability, while eight more are involved in 45%–75% of them (T5, F9, V11, D29, P31, L36, R38 and L50). These 19 residues mainly belong to alpha helices 1 and 2. This is not unexpected, since α-helices 2 and 2′ make up the rim and walls of the RBD pocket, the bottom of which mainly involves α-helices 1 and 1′ (Figure 1 and Figure 7a). Of note, D29, P31, F32 and to a lesser extent L36 are part of the high flexibility region that we identified at the N-terminus of helix α2 (see above). Some residues from α-helices 1 and 2 (e.g., K20, L33, D34, R44), having their side chains oriented towards the exterior of the structure as a result of the helical periodicity, are never involved in pockets.

Some additional residues are exclusively involved in highly druggable pockets and virtually absent from less druggable pockets: V18 and F22, and less frequently I64, V65, I68, T49 and F14. Most of the highly druggable pockets involve the combined participation of V18 and F22, even if in the crystal structure the latter is rather oriented at the exterior surface of the RBD. The joined implication of these two residues corresponds, however, to relatively accessible conformations, since it was observed in 81/323 pockets. Similarly, though less frequent, while residue F14 is, most of the time, exposed at the surface of the RBD, fluctuations that expose it to the interior of the groove result in highly druggable pockets. About two thirds of the group B pockets are highly druggable pockets that extend deeply into the hydrophobic pack of the RBD and also involve V65, I68 and less frequently I64.

**Figure A1:**
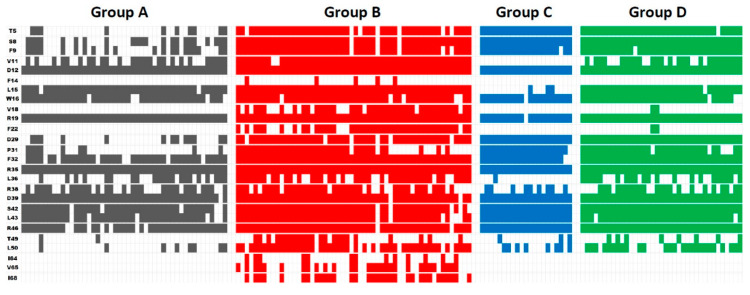
The most involved RBD residues and the four groups of pockets. Correspondence between the 26 most involved RBD residues (rows) and the 159 FL groove-pockets (columns). The selected residues are indicated on the left, and FL pockets are ordered and colored according to their group.

Figure 7b and supplementary Figure S3 show four pockets that are representative of the four groups (folder “pockets_4groups_PDB” in the supplementary data contain their PDB coordinates). The two groups of less druggable pockets (A and C, Figure 5) both involve low numbers of residues. Most of the group A pockets lie at the center of the groove and involve L15, which occupies a central position in helix α1, along with V11 and L36. To the contrary, group C pockets are generally asymmetric and positioned at one end of the groove. They distinctly involve three groups of residues: (i) T5, S8, F9 and D12 at the N-terminus of α1, (ii) R19 at the C-terminus of the antiparallel α1′, along with (iii) D29 and P31 that form the high-flexibility site at the N-terminus of helix α2′. Unlike pockets from groups A and C, those in groups B and D combine most of the above residue (i.e., T5, S8, F9, V11, D12, L15, R19) but also include F32, R35 and R46, reflecting their larger volume. They are also generally more symmetric than less druggable pockets, suggesting that they are broad pockets lying at the center of the groove and involving symmetric residues of the two monomers. The highly druggable, group B pockets distinctly involve the added participation of residues V18 and F22 combined, sometimes with the deeply buried residues I68 and V65 that confer the highest druggability scores (Figure 6). Figure A1 illustrates the participation of the 26 most involved residues to the four groups of pockets.

### 3.4. Most of the Residues Involved in the Druggable Pockets Are Strictly Conserved

**Figure 8:**
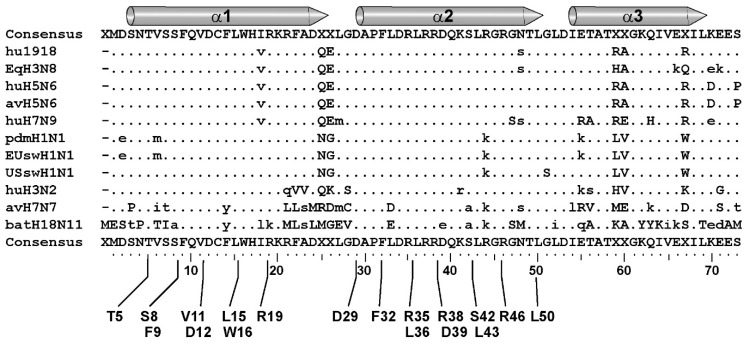
Conservation of the pocket-involved residues in the RBD. NS1 peptide sequences representative of human seasonal viruses H3N2 (huH3N2) and pdmH1N1, classical swine (USsw), Eurasian swine (EUsw), avian and human H5N6, human H7N9, equine H3N8, avian H7N7 and bat H18N11 were aligned using the “showalign” tool of software suite EMBOSS [55]. The three α-helices are represented by gray rods. Only differences relative to the consensus sequence (above and underneath) are shown (hyphens are deletions). The access numbers of the peptide sequences are as follows, in their order of alignment: AAK14368, AWW20412, AZN10062, QFP98359, QDK58640, QHA77971, AZN23118, AXC24890, QFZ88567, APC31570, AGX84939.

Finally, we wished to evaluate to what extent our results can be extrapolated to other sequence variants of NS1, since we performed the dynamics on only one representative structure corresponding to an individual peptide sequence of NS1. As shown in the sequence alignment (Figure 8), almost all of the residues that we found involved in the druggable pockets are strictly conserved over the diversity of NS1′s peptide sequence for the allele A of NS1, and most of them are also conserved in the distant sequences corresponding to allele B and to NS1 of the bat viruses. This is more specifically the case for T5, S8, F9, V11, D12, L15, W16, R19, F22 (V in currently circulating human H3N2 viruses), D29, F32, R35, L36, R38, D39, S42, L43, R46, T49, L50, and also V65 and I68.

## 4. Discussion

Our objective in this study was to systematically assess the flexibility of NS1′s structure and to take it into account in evaluating the druggable potential of its RNA-binding interface that encompasses the groove formed by the two antiparallel helices α2 and α2′. Indeed, by relying only on rigid crystallographic structures, one may miss most of the conformations that are sampled over the time by a breathing structure, including ones that can be targeted by small compounds. Pockets can transiently form as a result of local flexibility, and it is important to evaluate their physico-chemical and geometric properties to predict their druggability, i.e., their capacity to accommodate a drug candidate.

Using an MD approach and a representative three-dimensional structure of NS1, we first showed that NS1′s structure and particularly that of its RBD is remarkably stable, probably owing to the intricate network of interactions that were shown to stabilize its dimeric assembly [36]. However, monitoring the RMSF of the residues over the simulations allowed us to reveal a high-flexibility region, centered on residue P31 and encompassing the α1-α2 connecting loop and the N-terminal third of α-helix 2. There are two such sites in the dimeric RBD structure, symmetrically positioned at the two extremities of its long groove that is delimited by α-helices 2 and 2′ (Figure 7). Such a flexible behavior was not observed in the other loop that connects helices α2 and α3.

In the set of conformations that we sampled along the MD simulations, we searched for potentially druggable pockets, using our dedicated PockDrug server [31]. We specifically focused our search to the “groove region” that makes the RNA-binding interface of the RBD [33,41], based on the critical importance of the RNA-binding properties of NS1 on its multiple activities during the viral cycle [14,15]. Furthermore, the RBD has a highly conserved structure that is devoid of the conformational plasticity observed in the structure of the effector domain [16,17,56].

After the exclusion of the small-volume pockets involving fewer than 14 residues, our search identified 323 groove-pockets of variable druggability scores. Similar numbers of groove-pockets were identified in the FL- and RBD-set of conformations, suggesting that the transient formation of pockets by the RBD is a property inherent to its structure, regardless of the presence of the ED. It is noticeable that, unlike in the original crystal structure, most of the groove conformations (60%) observed along the dynamics were in a druggable state, i.e., they encompassed at least one pocket predicted as druggable by the PockDrug server. Indeed, the conformations that we sampled along the MD trajectories permanently oscillate between high and low druggable states. However, on average, in sixty percent of the sampled time points, the groove was in a druggable conformation.

Eighteen residues, mainly from α-helices 1 and 2, are involved in the majority of the pockets, regardless of their druggability score, and the latter generally increased with the number of residues involved. Ten residues (D12, W16, R19, F32, R35, D39, S42, L43, R46 and to a lesser extent R38) are similarly involved across the four groups of pockets, while eight more are consistently shared by the two groups of druggable pockets (T5, S8, F9, V11, L15, D29, L36, and L50). Druggable pockets (i.e., with a druggability score above 0.5) are large cavities lying at the center of the groove and involving symmetric residues of the two monomers. Those with the highest druggability scores involve, in addition, the combined participation of V18 and F22, and also extend deeper, up to residues V65 and I68.

All the residues involved in the groove-pockets are, most often, strictly conserved over the large sequence diversity of NS1. This probably relates to their critical role in both the structural properties and the biological activities of NS1. On the one hand, the highly stable structure of the dimeric RBD relies on several hydrophobic interactions, along with both intra-molecular (D12-R46, D29-R19) and inter-molecular interactions (D12-R35, D39-R35 and D29-R46) [36]. On the other hand, R38 and other residues that are exposed at the rim of the groove (R35, R37, R46) play a critical role in NS1 binding to RNAs [9,33,57,58,59]. Of note, the accessibility to the groove pockets may to some extent be modulated by the position of residue R38, which was shown to undergo a large conformational change upon RNA binding [33].

Previous studies have attempted to explore the druggable potential of NS1. Darapameni et al. [27] used Q-SiteFinder to search for potential Ligand Binding Sites in an approach that relied both on the volume of the pockets and on their binding energy to a virtual methyl group. The two main Ligand Binding sites that they identified as most promising in the RBD overlap with the pockets that we identified: the first involved D12, L15, W16, R19, P31-L33, R35, L36 and D39, while the second involved T5, V6, S8, F9, W16, R38, D39, S42, L43, R46 and L50. A third, deeper site involved some residues of α-helix 3 that we also identified. However, their approach was based on the analysis of rigid crystal structures, rather than on the dynamic simulations that we used. Our results confirm the interest of MD to understand the flexibility of a target and to identify its potential binding site(s) and the role of different residues in its druggability.

Our work confirms the interest of the groove-pockets as druggable sites that could be used to identify small compounds that specifically target the critical RNA-binding properties of NS1 and emphasize the value of NS1 as a promising target. Furthermore, the strict conservation of both the RBD structure and the residues involved in the druggable pockets across the large diversity of NS1 peptide sequences underlies the robustness of this search as a first step towards the identification of broadly active NS1-targeting compounds.

While other approaches based on biochemical or cell-based experiments have identified several compounds that specifically inhibit NS1 activities in cells [23,60] or that reduce viral replication in cultured cells [24], our work is a first step towards a complementary search for small NS1-targeting compounds by docking-approaches similar to those that were used to identify neuraminidase inhibitors [61,62].

## Supplementary Materials

The following are available online at https://www.mdpi.com/1999-4915/12/5/537/s1, Figure S1: Secondary structure map for FL and RBD simulations; Figure S2: Distribution of the four groups of groove pockets along the MDs; Figure S3: RBD conformations representative of the four groups of pockets; Table S1: The 17 pocket descriptors. PDB coordinates of representative pockets from the 4 groups.
