# Supplementary material for: Molecular Dynamics Simulations of Influenza A Virus NS1 Reveal a Remarkably Stable RNA-Binding Domain Harboring Promising Druggable Pockets
Source: Viruses. 2020 May 14;12(5):537. doi: 10.3390/v12050537 (PMC7290946; doi:10.3390/v12050537)
Supplement: Supplementary file 1 [file viruses-12-00537-s001.zip › viruses-784583-for conversion-suppl/viruses-784583_supplementaryData_13mai2020.pdf]

viruses

MDPI

Supplementary Data

Molecular Dynamics Simulations of Influenza A Virus NS1 Reveal a Remarkably Stable RNA-Binding Domain Harboring Promising Druggable Pockets

Hiba Abi Hussein [1,†], Colette Geneix [1,†], Camille Cauvin [1], Daniel Marc [2,3,*], Delphine Flatters [1,†,*] and Anne-Claude Camproux [1,†]

[1] Université de Paris, BFA, UMR 8521, CNRS, ERL U1133, Inserm, F-75013 Paris, France; hiba.abihussein@gmail.com (H.A.H.); colette.geneix@univ-paris-diderot.fr (C.G.); camille.cauvin@gmail.com (C.C.); anne-claude.camproux@univ-paris-diderot.fr (A.-C.C.)

[2] Equipe 3IMo, UMR1282 Infectiologie et Santé Publique, INRAE, F-37380 Nouzilly, France

[3] UMR1282 Infectiologie et Santé Publique, Université de Tours, F-37000 Tours, France

* Correspondence: daniel.marc@inrae.fr (D.M.); delphine.flatters@univ-paris-diderot.fr (D.F.)

1. Materials and Methods

Pocket descriptors. Pockets were first described using 75 descriptors that include the 52 descriptors developed by Borrel et al (2015) to characterise pockets (36 physico-chemical descriptors combined with 16 geometrical ones). After removal of the descriptors that are redundant or have a null variance, the 17 most informative descriptors that we retained are listed in the table below.

Supplementary Table S1: the 17 pocket descriptors

Name of pocket descriptors	
**Three descriptors of volume and shape**	
C_residues	Number of residues
Smallest size	Distance separating the two closest slabs enclosing the hull (Petitjean 1992) (RADI software)
PSI	Pocket Sphericity Index is the ratio of the radius of the largest sphere inscribed in the hull to radius of the smallest enclosing sphere. The closer PSI is to 1, the more spherical the pocket is. A small PSI value indicates that the pocket hull is flat. (Cerisier et al., 2017 ; http://petitjeanmichel.free.fr/itoweb.petitjean.freeware.html)
**14 physico-chemical descriptors**	
p_positive residues	Proportion of positively charged residues (H, K, R)
p_charged residues	Proportion of charged residues (D,E, R, K, H)
p_negative residues	Proportion of negatively charged residues (D, E)
p_polar residues	Proportion of polar residues (C, D,E, H, K, N, Q, R, S, T, W, Y)
Charge	Global charges (charged residues, aspartic acid, glutamic acid, lysine, arginine and ions) (Perola et al., 2012)
p_aliphatic residues	Proportion of aliphatic residues (I, L, V)
p_carbon atom	Proportion of carbon

p_main_chain atom	Proportion of atom main chain
p_small residues	Proportion of small residues (C, V, T, G, A, S, D, N, P)
p_tiny residues	Proportion of tiny residues (A, C, G, S)
T	Proportion of T
p_hydrophobic residues	Proportion of hydrophobic residues (C, G, A, T, V, L, I, M, F, W, Y, H, K)
V	Proportion of V
L	Proportion of L

2. Results

The analysis of the dynamic properties of wild-type RBD or FL dimer structures, for each of the three independent simulations, have shown low RMSDs (~ 2-2.5 Å) and preservation of the secondary structures over time, as shown in Figure S1 below.

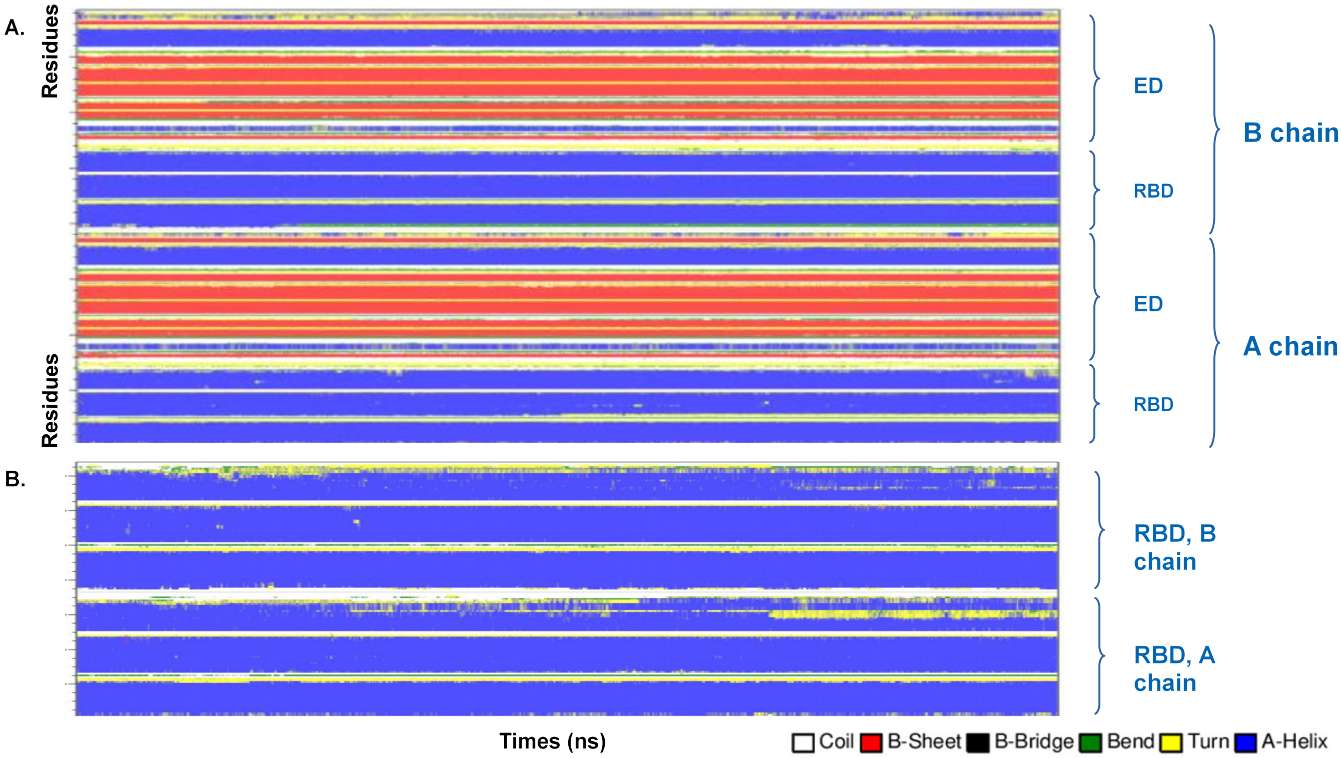

Figure S1. Secondary structure map for FL and RBD simulations. The secondary structures ((A) FL; (B) RBD) are represented for each residue (on the ordinate, chain A and chain B from bottom to top) over time (on the abscissa). The blue and red bands correspond to alpha-helices and beta-strands, respectively. Their conservation along the trajectories illustrates the stability of secondary structures along the simulations.

A

B

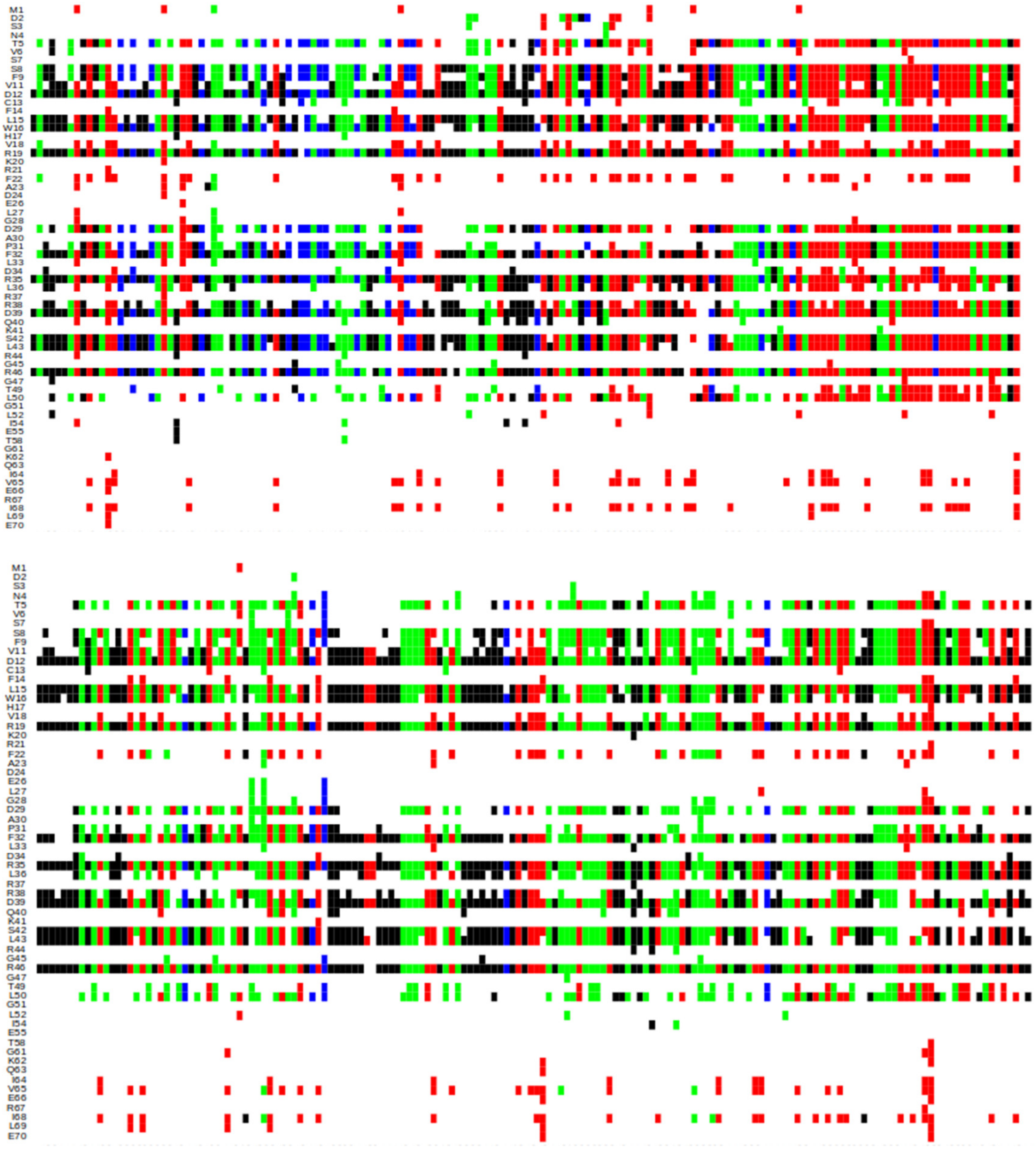

Figure S2. Distribution of the four groups of groove pockets along the MDs. Residues involved in each of the groove-pockets along the 3 independent MDs are represented in (A) the 159 groove-pockets over time in FL-conf and (B) the 164 groove-pockets over time in RBD-conf. Residues are shown in rows and groove-pockets in columns, colored according to their group (A in black, B in red, C in blue and D in green).

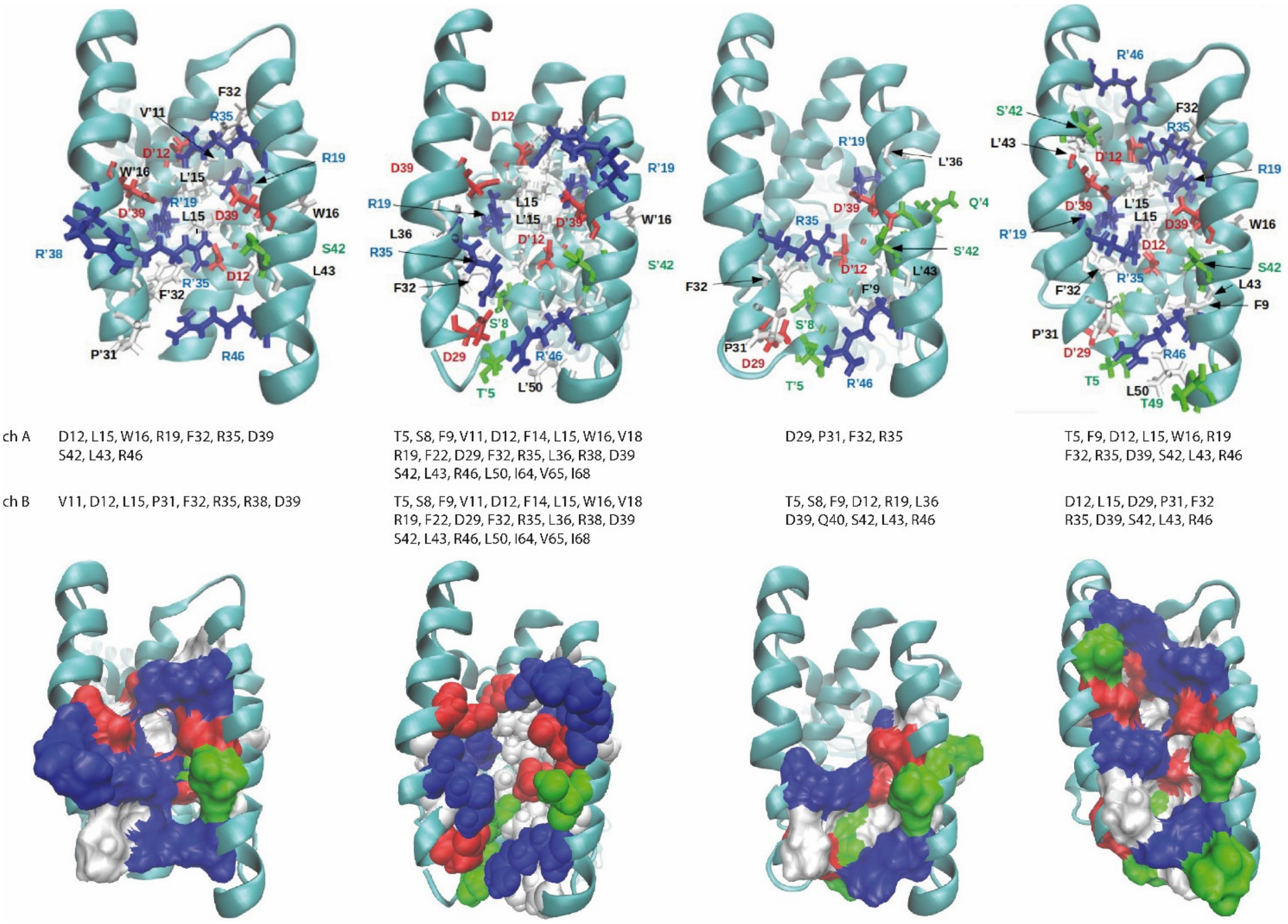

Figure S3. RBD conformations representative of the four groups of pockets. Conformations are in the order A, B, C, D. The amino acids (from chains A and B of the dimer) involved in the groove pockets are indicated below. Bottom: surface rendering of the corresponding pockets.

References

Borrel, A., Regad, L., Xhaard, H., Petitjean, M., Camproux, A.C. PockDrug: A Model for Predicting Pocket Druggability That Overcomes Pocket Estimation Uncertainties. *J Chem Inf Model* **2015**, *55*, 882-895.

Perola, E., Herman, L., Weiss, J. Development of a Rule-Based Method for the Assessment of Protein Druggability. *J Chem Inf Model* **2012**, *52*, 1027–103

Petitjean, M. Applications of the Radius-Diameter Diagram to the Classification of Topological and Geometrical Shapes of Chemical Compounds. *J Chem Inf Comput Sci* **1992**, *32*, 331–337.

Cerisier, N., Regad, L., Triki, D., Camproux, A.C., Petitjean M. Cavity Versus Ligand Shape Descriptors: Application to Urokinase Binding Pockets. *J Comput Biol* **2017**, *24*, 1134-1137.

© 2020 by the authors. Submitted for possible open access publication under the terms and conditions of the Creative Commons Attribution (CC BY) license (http://creativecommons.org/licenses/by/4.0/).
